# Myeloid leukocytes’ diverse effects on cardiovascular and systemic inflammation in chronic kidney disease

**DOI:** 10.1007/s00395-022-00945-4

**Published:** 2022-07-27

**Authors:** Alexander Hof, Simon Geißen, Kezia Singgih, Martin Mollenhauer, Holger Winkels, Thomas Benzing, Stephan Baldus, Friedrich Felix Hoyer

**Affiliations:** 1 Department III of Internal Medicine, Heart Center, Faculty of Medicine and University Hospital Cologne, University of Cologne, Cologne, Germany; 2 Department II of Internal Medicine and Center for Molecular Medicine Cologne, Faculty of Medicine and University Hospital Cologne, University of Cologne, Cologne, Germany; 3 Center for Molecular Medicine Cologne, University of Cologne, Cologne, Germany

**Keywords:** Myeloid cells, Cardiovascular inflammation, Chronic kidney disease, Infection, Systems immunology

## Abstract

Chronic kidney disease’s prevalence rises globally. Whereas dialysis treatment replaces the kidney’s filtering function and prolongs life, dreaded consequences in remote organs develop inevitably over time. Even milder reductions in kidney function not requiring replacement therapy associate with bacterial infections, cardiovascular and heart valve disease, which markedly limit prognosis in these patients. The array of complications is diverse and engages a wide gamut of cellular and molecular mechanisms. The innate immune system is profoundly and systemically altered in chronic kidney disease and, as a unifying element, partakes in many of the disease’s complications. As such, a derailed immune system fuels cardiovascular disease progression but also elevates the propensity for serious bacterial infections. Recent data further point towards a role in developing calcific aortic valve stenosis. Here, we delineate the current state of knowledge on how chronic kidney disease affects innate immunity in cardiovascular organs and on a systemic level. We review the role of circulating myeloid cells, monocytes and neutrophils, resident macrophages, dendritic cells, ligands, and cellular pathways that are activated or suppressed when renal function is chronically impaired. Finally, we discuss myeloid cells’ varying responses to uremia from a systems immunology perspective.

**Supplementary Information:**

The online version contains supplementary material available at 10.1007/s00395-022-00945-4.

## Clinical relevance of chronic kidney disease for cardiovascular disease

Chronic kidney disease (CKD) is defined as abnormalities of kidney structure or function, present for more than 3 months, particularly in patients with impaired glomerular filtration rate (GFR < 60 ml/min/1.73m^2^) [63, 145]. CKD is an increasingly prevalent condition and affects approximately 15% of the adult population worldwide [14, 42, 114, 129, 132]. The international Kidney Disease: Improving Global Outcome organization categorizes disease severity based on cause, GFR and level of albuminuria. Three stages, based on the degree of albuminuria, and five stages, dependent on the GFR, are distinguished [62, 63]. CKD’s early to intermediate stages dominate the prevalence statistics [49]. Causes for CKD are manifold, yet traditional cardiovascular risk factors such as male sex, age, hypertension, hyperlipoproteinemia and diabetes also propel CKD progression. Further, genetic and epigenetic mechanisms impact CKD development [145].

**Fig. 1:**
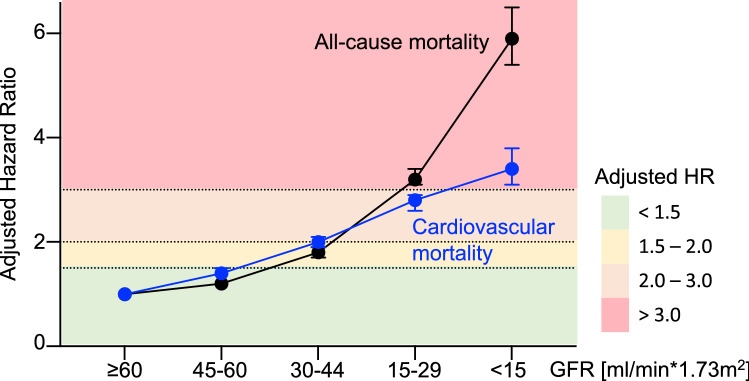
CKD elevates all-cause and cardiovascular mortality. The adjusted hazard ratios (HI) for all-cause mortality (black line) and cardiovascular mortality (blue line) increase in a GFR-dependent manner in patients with CKD. Data for this graph are adopted from Go et al., NEJM 2004 [42]

The Global Burden of Disease Study estimates that CKD caused at least 1.2 million deaths in 2017 [32]. Concomitant cardiovascular disease and an elevated susceptibility for infections fuel mortality’s sharp increase. Indeed, mortality rises significantly and in a stage-dependent manner when CKD is present. In a meta-analysis including > 14 million participants from 14 different studies, all-cause mortality risk was unrelated to a GFR between 75 and 105 ml/min but associated to lower GFR with an adjusted hazard ratio at GFR 60, 45, and 15 ml/min of 1.18 (95% confidence interval CI 1.05–1.32), 1.57 (CI 1.39–1.78) and 3.14 (CI 2.39–4.13), respectively, as compared to normal kidney function (GFR 95 ml/min) [14]. Similar results were seen in an integrated system of health care analysis including data from 1.120.295 adults (Fig. 1) [42]. In this study, the adjusted hazard ratio for death was 1.2 (CI 1.1–1.2) with a GFR of 45–59 ml/min, 1.8 (CI 1.7–1.9) with a GFR of 30–44 ml/min, 3.2 (CI 3.1–3.4) with a GFR of 15–29 ml/min and 5.9 (CI 5.4–6.5) with a GFR of less than 15 ml/min. Accordingly, the same cohorts displayed inversely increased, adjusted hazard ratios for cardiovascular events with 1.4 (CI 1.4–1.5), 2.0 (CI 1.9–2.1), 2.8 (CI 2.6–2.9) and 3.4 (CI 3.1–3.8), respectively [42].

CKD severity correlates with the increase in cardiovascular risk [70]. In the National Health and Nutritional Examination Survey (NHANES), cardiovascular disease burden was investigated in 1428 CKD stage I-IV patients between 2001 and 2010. In this cohort, the prevalence of cardiovascular co-morbidities rose markedly, with 19.6% having coronary artery disease, 10.3% having a stroke, 9.7% congestive heart failure, and an overall risk for cardiovascular disease of 28.4% [70]. Other investigations also showed an increase in overall risk for cardiovascular disease of 40% in CKD patients and, as expected, cardiovascular disease was most pervasive in CKD stage IV [13]. Renal replacement therapy associates with a tremendously risen (~ 30-fold) mortality risk [70]. Consequently, cardiovascular mortality is about 57% higher when CKD is present [13, 22, 42, 104]. Importantly, patients suffering from CKD are more likely to die from cardiovascular co-morbidities than from end-stage renal failure, even after adjustment for cardiovascular risk factors [18, 42, 61]. Whereas CKD and cardiovascular disease share multiple risk factors, statin therapy fails to curb cardiovascular events in end-stage renal failure [143]. Although pharmacotherapies that alter intrarenal hemodynamics (e.g. renin–angiotensin–aldosterone pathway modulators and SGLT2 inhibitors) can preserve kidney function by reducing intraglomerular pressure and novel antifibrotic agents have the potential to retard disease progression, no specific treatments are yet available mitigating CKD’s risk on the vasculature. Moreover, atypical symptoms or the lack of cardinal clinical signs in CKD may further delay timely treatment [111].

Aside from vascular disease, numerous studies associate hemodialysis treatment in end-stage renal disease with accelerated aortic valve calcification and stenosis development. More than half of patients on renal replacement therapy display aortic valve pathologies, as assessed by computed tomography scans in a study with 151 patients [10]. Evidence emerges that aortic valve remodeling may already occur in the early stages of kidney failure in a GFR-dependent manner [10, 46, 133]. Mortality rises markedly in patients with aortic valve stenosis that undergo aortic valve replacement surgery, when CKD is present. In a multi-center study including data from the German Aortic Valve Registry, CKD’s impact on mortality risk was investigated in nearly 30,000 patients from 88 centers undergoing surgical aortic valve replacement or transcatheter aortic valve implantation (TAVI). One-year mortality hazard ratios increased gradually with declining renal function after transcatheter aortic valve implantation, ranging from 1.22 in CKD stage III to 3.95 in CKD stage V, compared to patients in CKD’s early stages [83]. In summary, CKD is an independent risk factor for cardiovascular and valvular disease.

End-stage renal failure engenders a susceptible environment for infections; bloodstream infections and pneumonia are the second most common cause of death in patients with CKD. Accordingly, mortality risk due to infections rises tremendously, up to 50-fold, in end-stage renal failure [116]. In analogy to cardiovascular disease, even mild to moderate stages of kidney disease raise infection rates and subsequent mortality [56, 142].

In a cohort of 25.675 patients, the risk for bloodstream infections increased GFR-dependently with hazard ratios of 1.24 (1.01–1.52), 1.59 (1.24–2.04) and 3.54 (2.69–4.69) when compared to individuals with a GFR above 60 ml/min for CKD stage IIIa, IIIb, and IV, respectively. In turn, community-onset bloodstream infections elevate the risk of death within 30 days in patients with CKD stage IV or below (hazard ratio, 4.10; 2.06–8.14) [58]. Whereas CKD’s advanced stages correlate with the risk of infection, distinct co-morbidities may impact the threshold for infections in the early stages [93].

Together, abundant clinical studies provide evidence for CKD’s perilous role in cardiovascular disease development. CKD is accompanied by a malfunction of the immune system affecting leukocyte interactions and activity on a cellular and subcellular level. We will review how a dysfunctional immune system accelerates vascular and valvular disease progression in CKD. Systemic effects on the contrary interfere with anti-bacterial defense mechanisms. Tissue and cell type may decisively determine these diverse effects on the immune system. Here, we provide a summary of the immune system’s facets in CKD’s two most important complications.

## Chronic kidney disease impacts myeloid cell behavior in atherosclerosis

### Uremic toxins alter myeloid cells in chronic kidney disease

The innate immune system comprises different cell types. Major circulating contenders include neutrophilic granulocytes with a short life span of approximately one day. Blood monocytes are less frequent in mice and humans but exhibit a longer life span ranging from days to a few weeks [89]. The use of elaborate mouse models has greatly contributed to the refined understanding of myeloid cell biology in steady-state and disease in the last two decades: blood myeloid cell levels fluctuate in a circadian pattern and are replenished by bone marrow supply at all times [89]. Tissue-resident macrophages, which populate various tissues prenatally–independent of definitive hematopoiesis–are myeloid cells [25, 36]. They are more long-living than their circulating comrades, and proliferation significantly maintains population size in the steady-state in various organs. Bone marrow-derived, circulating monocytes infiltrate tissues and give rise to macrophages ubiquitously in an acute or chronic injury [89]. Whereas monocyte recruitment feeds the inflammatory myeloid cell pool in atherosclerosis in the early stages, macrophage (i.e., foam cell) proliferation prevails and expands the vascular population in advanced stages [89]. Neutrophils engender atherosclerotic plaque instability by employing eroding enzymes or worsen the ischemic injury by forming extracellular traps (NETs) [52]. CKD amplifies existing, harmful pathways evoked by risk factors such as diabetes and hypercholesterolemia or–worst case–activates orthogonal mechanisms accelerating atherosclerosis (Suppl. Table 1). Phenotypically, excessive calcification is a typical feature of atherosclerotic lesions in CKD [7].

**Fig. 2:**
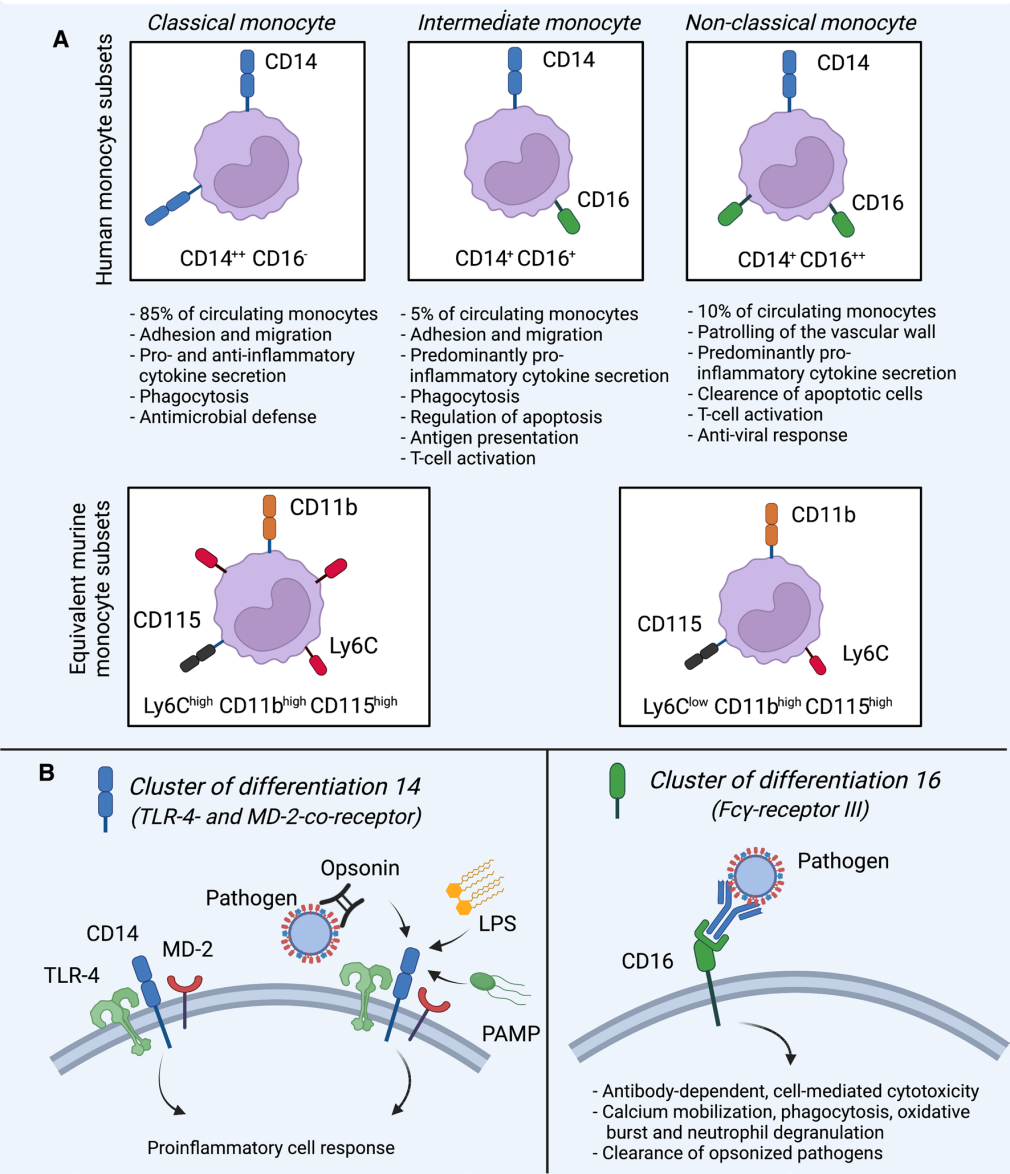
Human and murine monocyte subsets. **A** Human monocytes can be categorized by surface expression of CD14 and CD16 into classical, intermediate and non-classical monocytes [60, 90, 147, 154]. Classical monocytes do not express CD16. They are the most frequent subtype, mediate antimicrobial defense and display a high capacity of adhesion, migration and phagocytosis [60, 90]. Intermediate monocytes make up only ~ 5% of the population [112]. They express CD16 and are involved in regulation of apoptosis, antigen presentation and T-cell activation [60]. Non-classical monocytes express higher levels of CD16 than intermediate monocytes, do not extravasate but patrol the vessels [127]. They are involved in Fc-Receptor-mediated phagocytosis, anti-viral response and T-cell activation [60, 90, 112, 127, 147, 154]. In mice, equivalent monocyte subsets are identified by surface expression of Ly6C (usually by flow cytometry), whereas all monocytes express CD11b (an integrin) and CD115 (the macrophage colony-stimulating factor-1 receptor). Ly6C^high^ monocytes are considered equivalent to classical monocytes and Ly6C^low^ monocytes to the non-classical subset [16, 86]. **B** CD14 is a co-receptor for toll-like receptor-4 (TLR-4) and myeloid differentiation factor-2 (MD-2). CD14 facilitates sensing of lipopolysaccharide, pathogen-associated molecular patterns (PAMPs) and opsonized particles. The cellular response is generally pro-inflammatory [149]. CD16 (Fcγ-receptor III) mediates antibody-dependent cytotoxic effects, clearance of opsonized pathogens and fosters calcium mobilization, ROS-release and phagocytosis [151]

Monocytes isolated from uremic patients exhibit various signs of activation. They display increased adhesiveness, which fosters extravasation through the endothelial barrier, and an augmented migratory capacity [8]. While this study only included 28 patients, other studies corroborate CKD’s significant effects on monocytes in humans. For instance, CKD goes along with elevated numbers of CD14^+^ CD16^+^ monocytes, also known as the intermediate monocytic phenotype in humans, associated with endothelial injury and future cardiovascular events [90, 107, 110]. For an overview of human monocyte subtypes, see Fig. 2. A subtype of intermediate monocytes displaying high levels of the human leukocyte antigen (HLA) -DR enriches in patients with CKD stage I to V and correlates with renal function, i.e. GFR, as shown in a study involving 187 patients. Likewise, granulocytic neutrophils inversely correlate with renal function [90]. Ex-vivo, a uremic environment nudges monocytes from healthy donors towards a more inflammatory state and induces surface CD14 and CD16 expression [9]. While ex-vivo and in-vitro studies must be interpreted cautiously as cells’ transcriptional program adapts promptly to an altered exterior and the study’s sample size was small, these findings suggest an immediate immune-altering effect initiated by the uremic milieu. In this context, expression of the C–C chemokine receptor-2 (CCR2), which mediates monocyte recruitment and homing, rises in patients undergoing hemodialysis [96]. In this study, which included 83 patients, CCR2 levels correlate with markers of atherosclerosis, such as the carotid intima-media thickness. Uremia may also drive monocytic CX_3_CR1 expression–the fractalkine receptor facilitates adhesion to the endothelium–with consequences for monocyte homing, as demonstrated in a small study examining blood monocytes from patients receiving renal hemodialysis [21, 118]. Other studies found augmented toll-like receptor (TLR) -2 and -4 expression on blood monocytes in patients with CKD, rendering them more sensitive to consecutive inflammatory stimuli [45, 66]. Uremic signaling, however, is not restricted to a single route. Various pathways may be activated and mediate uremia’s effects, such as the inflammatory Wnt/β-catenin cascade in patients with CKD stage IV and V [2]. Post-translational protein modifications, such as acetylation of the Y-box binding protein-1 in monocytes, may further amplify inflammation, when hemodialysis is required [26]. There is ample evidence that CKD impacts myeloid cell behavior, however, some of these studies’ small sample sizes should be considered for interpretation and generalizability.

Considering the plethora of pathways involved, the question arises of what stimuli initiate inflammation in uremia. In CKD, extensive mineral remodeling involves osteoblasts and osteocytes, the primary source of fibroblast growth factor-23 (FGF-23). The 32-kDa hormone level rises in CKD’s early to advanced stages. Indeed, FGF-23 drives macrophage proliferation via FGF receptor-1c and instigates tumor necrosis factor (TNF)-α generation in-vitro, linking bone remodeling to myeloid cell inflammation in CKD [67, 84]. Next to FGF-23, much effort has been spent to elucidate the role of another large group of potential mediators: so-called uremic toxins are small molecules that evade hemodialysis by binding larger-sized plasma albumin. Dozens of uremic retention solutes have been categorized to date, indoxyl sulfate and p-cresyl sulfate are among the best-studied (Table 1). Effects are not confined to white blood cells, as uremic toxins also hamper the endothelium, vascular smooth muscle cells, and others. But the evidence for wide-ranged, immune-stimulatory effects is vast:

**Table 1:**
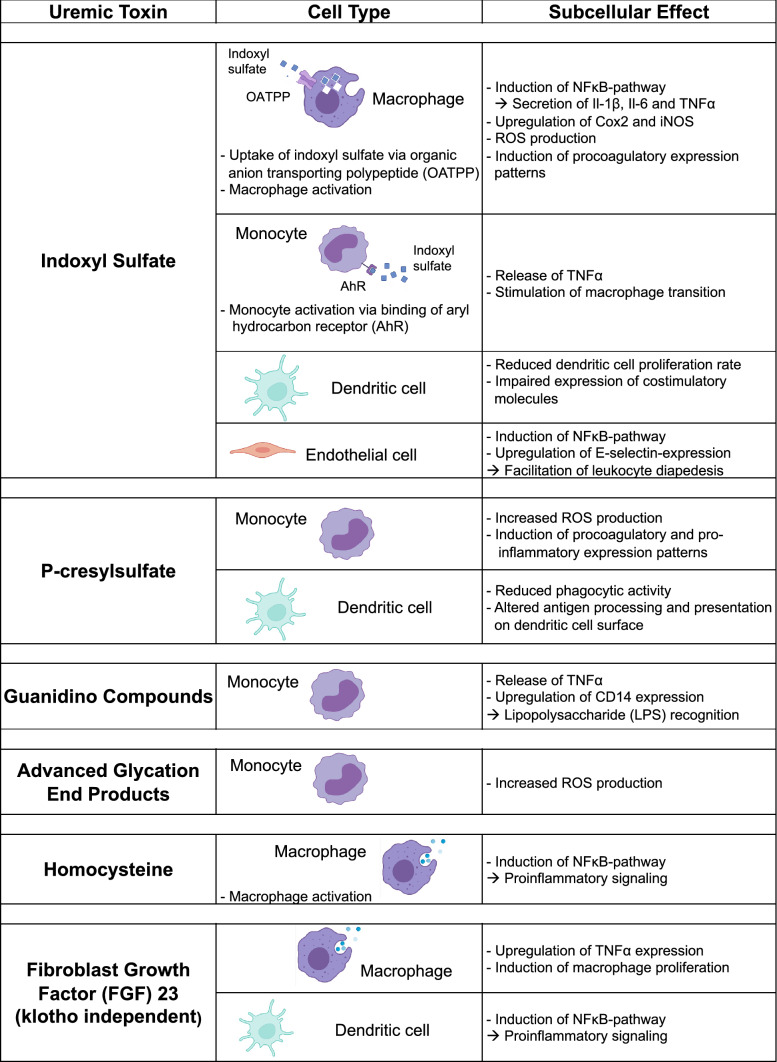
Effect of uremic toxins on different myeloid cell types

Indoxyl sulfate is transferred to the intracellular space of macrophages by the organic anion transporting polypeptide (OATPP) and induces pro-inflammatory cell activation by inducing the NFκB-pathway. Consecutively, Interleukin- 1β, Interleukin-6, Tumor Necrosis Factor-α (TNF-α) and reactive oxygen species (ROS) are released. Cyclooxygenase-2 (COX2), the inducible Nitric-Oxide-Synthase (iNOS) and pro-coagulatory factors are upregulated. Monocytes are activated by indoxyl sulfate via the aryl hydrocarbon receptor, which propels proinflammatory cytokine release, while dendritic cell proliferation and signaling is suppressed by indoxyl sulfate. As for macrophages, the NFκB-pathway is also induced in endothelial cells and adhesion molecules like E-selectin are upregulated, facilitating diapedesis. P-cresylsulfate induces ROS-production and pro-inflammatory expression patterns in monocytes. In dendritic cells, it reduces phagocytic activity and antigen presentation. Guanidino compounds such as creatinine or guanidine and advanced glycation end products increase ROS and TNF-α production as well as CD14 expression in monocytes. Homocysteine and fibroblast growth factor (FGF)-23 affect macrophages and dendritic cells by induction of pro-inflammatory signaling and proliferation. Interestingly, for FGF-23 these mechanisms are independent of its co-factor klotho

For instance, guanidino compounds induce TNF-α secretion and CD14 expression in monocytes in-vitro. Atherogenic homocysteine activates the NF-ĸB pathway in THP-1-derived macrophages in cell culture experiments [41, 117]. A very robust study conducted by Nakano and colleagues demonstrated that organic anion transporting polypeptides facilitate indoxyl sulfate uptake into macrophages. Consecutive NOTCH signaling triggers the release of pro-inflammatory interleukin (IL)-1β, an effect attenuated by delta-like ligand 4 inhibition in-vitro [92]. The use of nanoparticle-mediated silencing of the organic anion transport polypeptide in mice demonstrates the pathway’s relevance also in-vivo.

Another study confirms uremic toxins’ inflammatory effects in the vasculature ex-vivo. A brief exposure of rats to indoxyl sulfate or p-cresyl sulfate for four days led to profound inflammatory and pro-coagulatory signatures in aortic tissue, as assessed by an unbiased quantitative proteomics approach followed by gene ontology analyses. Acute-phase signaling pathways dominated in the analyses [99]. The underlying cell types driving inflammation in this setting remain elusive.

Finally, accumulation of p-cresyl sulfate and advanced glycation end-products ramp up reactive oxygen species generation in monocytes, as shown in ex-vivo and in-vitro experiments [40, 119]. Similar observations were made in neutrophils. When isolated from patients with CKD and subsequently challenged with PMA (phorbol-12-myristate-13-acetate), reactive oxygen species release amplified, an effect mainly conveyed by uremic toxins. Thus, neutrophil priming is central to low-grade inflammation and oxidative stress surplus in CKD, which occurs before renal replacement therapy is required [17, 122].

The question arises by which subcellular mechanisms uremic toxins trigger the litany of inflammatory responses [38]. While uremic toxins indirectly promote inflammatory gene transcription, e.g. via NOTCH signaling, binding to post-translationally modified proteins likely contributes to systemic inflammation. Further mechanisms probably exist but remain to be discovered [28, 85, 92].

### Compromised gut integrity amplifies cardiovascular inflammation in CKD

While the role of uremic retention solutes has garnered much attention, it recently emerged that compromised remote tissues in CKD impact inflammation and myeloid cell function. For instance, an impaired gut barrier function has been described, which is accompanied by profound changes in the intestinal microbial flora composition [134, 146]. The mechanisms perturbing the intestine’s barrier function remain incompletely understood [113]. Next to metabolic acidosis, volume overload with consecutive wall congestion conceivably contributes to the gut’s leakiness in CKD, as described for heart failure [43]. Distinct strains’ high pathogenicity–so-called pathobionts–such as *Bacteroides, Paraprevotella spp., or Helicobacter hepaticus* pose a particular threat [4]. Overgrowth of potentially pathogenic bacterial species is frequent in patients undergoing hemodialysis, and the disrupted commensal bacterial flora generates surplus trimethylamine-N-oxide (TMAO) [125, 150]. The microbiota metabolite not only correlates with cardiovascular risk. TMAO signals via macrophage CD36-dependent MAPK/JNK-pathway and promotes atherosclerosis in mice [33]. The leaky and permeable gut barrier facilitates bacteria translocation to extra-intestinal sites such as mesenteric lymph nodes, spleen and liver, and blood levels of bacterial DNA associate with serum levels of C-reactive protein (CRP) and IL-6 [141, 146]. Following translocation, bacteremia and endotoxemia may instigate systemic inflammation, yet, clear evidence that bacterial elements in CKD augment myeloid cell-driven vascular inflammation is still missing. In the steady-state, microbiota-derived peptidoglycan primes bone marrow neutrophils via the pattern recognition receptor nucleotide-binding, oligomerization protein-1 (Nod1) and augments systemic immunity [15]. While desirable for host defense, this priming may conceivably be harmful for the inflamed vasculature. Whether this mechanism, however, is compromised in CKD remains unclear. Furthermore, the ramification may not be entirely inflammatory, as prolonged toll-like receptor activation can contribute to immunosuppression [4].

### Myeloid cells’ catalyzing role in CKD-driven vascular inflammation

**Fig. 3:**
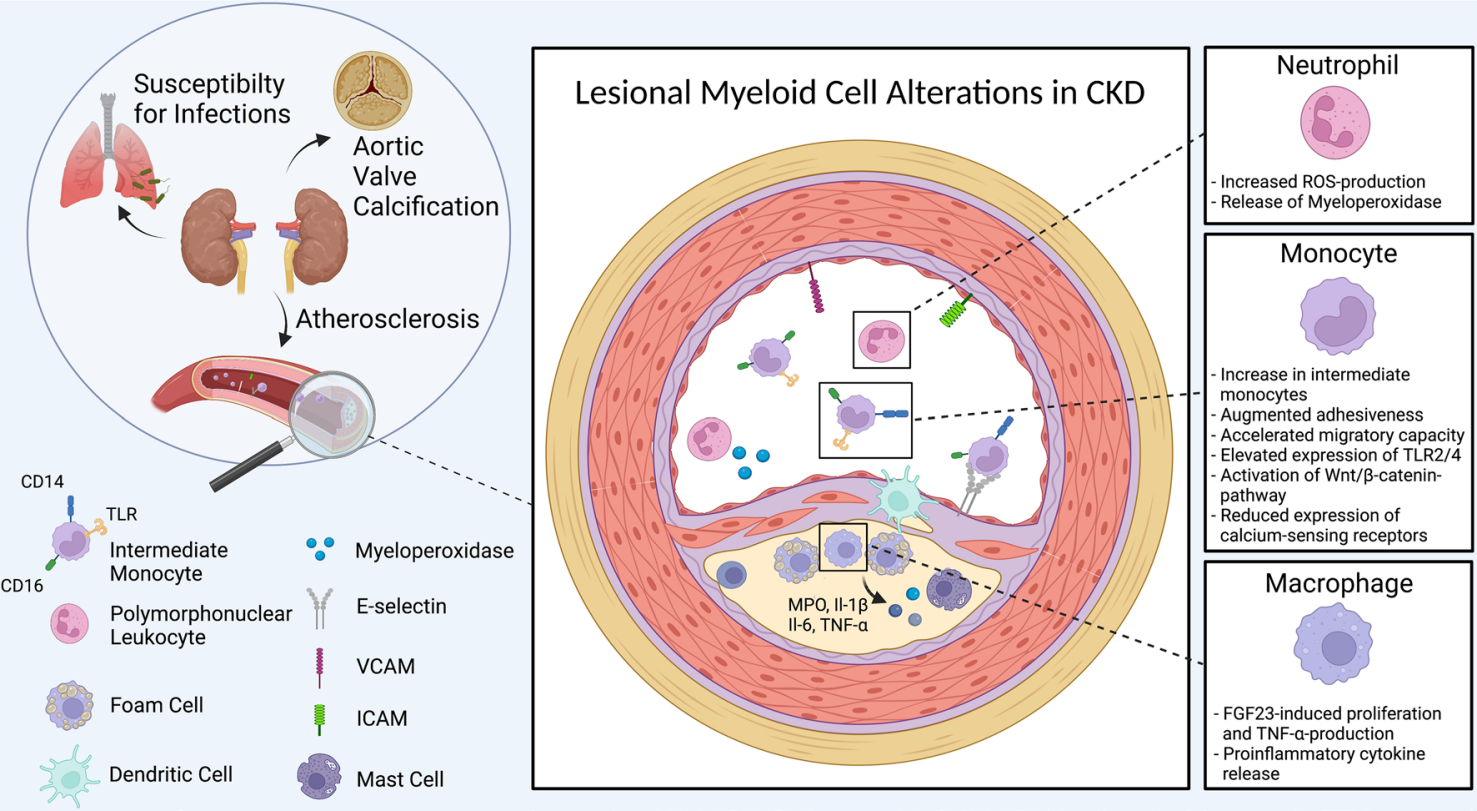
Remote organ complications in chronic kidney disease. Myriad remote organ pathologies accompany CKD and increase the susceptibility to infections, the incidence of aortic valve calcification, and atherosclerosis [10, 56, 70]. Alterations at the site of lesion formation include augmented ROS production of neutrophils, an increase of intermediate monocytes with enhanced adhesiveness and migratory capacity, and elevated TLR-2 and -4 expression [17, 45, 66, 90]. The WNT/β-catenin pathway is involved [2]. CKD lowers the expression of monocytic calcium-sensing receptors, which may accelerate the vessel wall’s calcification [82]. Under uremic conditions, macrophages’ and foam cells’ proliferation rates increase [94]. Likewise, pro-inflammatory cytokine secretion amplifies [11, 101]. The endothelium cranks up expression of adhesion molecules such as E-selectin, VCAM and ICAM in response to uremia [11, 57, 101.

In light of atherosclerosis’ chronic inflammatory nature and myeloid cells’ aberrant features in CKD, a multitude of studies examined their contribution to vascular disease progression in experimental CKD. For an overview, please see Fig. 3. As described for traditional cardiovascular risk factors, CKD may trigger endothelial signaling cues guiding monocytic leukocyte influx into the arterial wall. For example, levels of circulating VCAM-1 and ICAM-1 increase in patients receiving hemodialysis, as demonstrated in a study involving 106 patients [101]. The surge’s cause is not entirely clear but likely reflects the enhanced vascular inflammation. In-vitro, indoxyl sulfate signals via the c-Jun N-terminal kinase pathway inducing expression of E-selectin in endothelial cells [57]. Therefore, CKD-mediated signs of vascular activation are partly redundant (Suppl. Table 1) [11, 57, 101]. The uremic milieu further impairs the glycocalix’ integrity, the physiologic layer of proteoglycans and glycoproteins covering the endothelium’s luminal surface, as serum markers reflecting glycocalix injury rise in patients with CKD. The altered glycocalix composition may in turn facilitate monocyte extravasation [75, 108]. In response to macrophage- and granulocyte–macrophage-colony stimulating factor (M-CSF; GM-CSF) secreted by the endothelium and other vessel wall cell types, monocytes differentiate into macrophages [39]. Increased M-CSF levels in hemodialysis patients may fuel macrophage development once monocytes infiltrate the nascent lesion [94]. However, the source for M-CSF’s surplus in this setting requires further investigations and experimental evidence for this mechanism is still missing [94]. While GM-CSF treatment exacerbates atherosclerosis in non-uremic mice, it remains unclear whether GM-CSF promotes vascular inflammation in CKD. GM-CSF’s role in CKD has been investigated in the context of vaccination, which we discuss below [47].

A perturbed equilibrium generates an excess of reactive oxygen species in CKD, which impacts atherosclerosis and the underlying myeloid cell population. CKD’s enabling effects on reactive oxygen molecules enhances low-density lipoprotein oxidation, consecutively, lesional macrophages’ inflammatory profile amplifies [27, 39, 48]. Urea dissociation creates reactive isocyanic acid which non-enzymatically and irreversibly binds protein amino-groups, especially lysine residues. This process–termed carbamylation–promotes molecular ageing and associates with atherosclerosis [44, 135]. The heme enzyme myeloperoxidase (MPO) augments carbamylation through the oxidation of thiocyanate. Myeloperoxidase also converts low-density lipoprotein into a more atherogenic form, as shown in elaborate experiments in-vitro [105]. In a CKD mouse model using atherosclerosis-prone LDL receptor-deficient mice, lesional macrophages enrich myeloperoxidase and the enzyme’s activity heightens, suggesting a disease-promoting role via carbamylation [68, 153]. Future experiments using–currently unavailable–MPO reporter or conditional knockout mouse models will allow to obtain more insights into MPO’s role in CKD-driven vascular inflammation. Finally, the increase in myeloperoxidase and distinct uremic metabolites such as asymmetric dimethylarginine diminish nitric oxide (NO) bioavailability with consequences for the endothelium’s function in patients with CKD [19, 78].

Additional myeloid lineage cells may promote atherogenesis in CKD. Mast cells increasingly accumulate in the shoulder and basis of atherosclerotic plaques when CKD is present but do not associate with calcification [139]. In a small case–control study examining human aortic tissue samples of 10 individuals, dendritic cells enriched in the tunica intima, the innermost vascular layer, in patients with CKD [54]. Yet, a detailed understanding of dendritic and Mast cells’ relevance in CKD-driven atherosclerosis is lacking. Activation of CD4 and CD8 T-lymphocytes conceivably plays a role, as demonstrated for vascular inflammation without concomitant CKD. It should be noted that non-inflammatory factors such as phosphate and calcium deposition may promote atherosclerosis progression in CKD, emphasizing the processes’ multitude.

Numerous clinical studies provide compelling findings for augmented vascular inflammation in CKD. Coronary vessels exhibit a greater extent of plaque formation with an increased local inflammatory milieu in CKD [91, 115]. Urea plasma levels and glomerular filtration rates are independent predictors of arterial wall inflammation [8]. Elevated serum levels of CRP, IL-6, TNF-α, and monocyte chemotactic protein-1 accompany heightened atherosclerotic plaque inflammation in patients with end-stage renal failure [11, 101]. The neutrophil-to-lymphocyte ratio correlates with the cardiovascular risk profile in patients with CKD [97]. A subset of low-density granulocytes associates with vascular calcification in patients receiving peritoneal dialysis [109]. While these clinical studies did not exclusively focus on myeloid cells, they feed the assumption for inflammation’s causative role in CKD-accelerated atherosclerosis.

## Chronic kidney disease impacts aortic valve remodeling and inflammation

Tricuspid aortic valves consist of three annulus-attached, semilunar cusps. Human aortic leaflets are thin (~ 180 µm in diameter) yet no simple structures. Five connective tissue layers build the leaflets’ backbone, which is interspersed with mesenchyme-originating interstitial cells. Endothelium covers and protects the leaflets’ surface [87]. Recent findings further indicate the existence of a valve-resident leukocyte population, as CD45-positive leukocytes prenatally populate the endocardial cushion of developing valves as early as embryonic day 14.5 in mice [5]. CCR2-expressing macrophages are present shortly after birth. Mapping valve leukocyte fate revealed an increasing accumulation of CD45-positive cells in intact murine valve tissue over time [5]. At the age of 16 months, approximately 11% of the examined murine valvular cells were leukocytes. CD45-positive cells predominantly accumulate at the cusps’ distal tips but also adjacent to the ventricular layer in young and adult mice. Lineage tracing experiments suggest that myeloid origin i.e. macrophages and dendritic cells dominates the heart valve leukocyte population [55].

Aortic valves are subjected to a humongous workload; they open and close ~ 86.000 times per day and ~ 2.5 billion times by the age of 80 years (based on an average heart rate of 60 beats per minute). Hence, the decades-prevailing view that passive mechanisms, i.e., continuous exposure to physical force and ectopic mineral deposition ultimately evoke valve degeneration. More recent insights shifted this paradigm suggesting leaflet remodeling and calcification involves activation of resident interstitial, endothelial and inflammatory cells [77, 124, 144]. Attempts to reseed valvular matrices with interstitial and endothelial cells may reflect this redirected perception [51, 74]. That inflammatory mechanisms impact cusp remodeling is now increasingly appreciated: TLR-3 signaling induces an osteogenic response in valvular interstitial cells in-vitro. Likewise, myeloid cell supernatant promotes osteogenic differentiation of interstitial cells in-vitro [73]. Disruption of anti-inflammatory IL-1-receptor signaling fuels inflammation–especially in the leaflet’s lamina spongiosa–and triggers subendothelial macrophage accumulation in mice. IL-1β, the prototypical inflammatory, macrophage-derived cytokine, amplifies remodeling via myofibroblast activation [123]. The TNF-α related ligand TRAIL may foster human valve calcification [29]. NOTCH1 signalling’s role for congenital valve anomalies and calcification is well-established [30]. That NOTCH1 haploinsufficiency alters macrophage major histocompatibility complex (MHC) II expression levels in aortic valves implies that inflammation potentially contributes to NOTCH1’s striking phenotype [106]. Examining aortic valves obtained from 285 patients undergoing surgical valve replacement revealed the presence of chronic inflammatory infiltrates in 28% of the cases. Intriguingly, inflammatory infiltrates correlated with the remodeling process and the peak transvalvular gradient [20].

CKD propels premature valve calcification and stenosis. The process is multilayered; plenty mechanisms are suspected partaking in uremia-induced valve remodeling: endothelial dysfunction due to CKD fluid overload-related heightened shear stress, lipid infiltration (though statin therapy is ineffective), reactive oxygen species surplus, bone metabolism dysregulation, calcium-phosphate imbalance and ectopic calcification, to name a few [128]. Data on how CKD shapes the inflammatory environment within the valve on the contrary are relatively scarce compared to vascular pathologies; and whether the same pathways are involved as to when CKD is not compromised is largely unknown. In a recent histologic study examining human stenotic aortic valve specimens, macrophages especially clustered in areas of valve calcification. While 8.3% of these patients received hemodialysis treatment, information whether early stages of CKD were present in the other individuals is missing, thus CKD’s influence was likely underestimated [95]. On a systems level, inflammation in CKD associates with the development of aortic valve stenosis. As such, CRP levels strongly correlate with valve calcification, as shown in a study involving 137 patients treated with continuous ambulatory peritoneal dialysis [140]. In a study involving 55 patients receiving hemodialysis treatment, aortic stenosis occurred in 14 individuals and significantly correlated with heightened CRP levels [120]. Likewise, IL-6 levels associate with the risk for valvular calcification, as demonstrated in a cross-sectional study involving 135 patients with CKD and 58 control individuals [72]. Another recent, interesting observation at the hub of inflammation and calcification may become especially relevant for valvular pathologies. CKD associates with significantly reduced expression levels of G-protein-coupled calcium-sensing receptors on isolated monocytes. In-vitro, the decrease in calcium-sensing receptor expression impaired monocytes’ ability to inhibit vascular calcification [82]. In summary, evidence emerges that inflammation impacts valve remodeling in CKD, yet a detailed understanding of myeloid cells’ contributions to this disorder is still lacking.

## Chronic kidney disease impairs myeloid cells’ responses to infection

The organism’s susceptibility for bacterial infections rises dramatically whenever kidney function fails–especially when renal insufficiency mandates replacement therapy. Infectious disease claims the second most casualties in CKD [101, 126]. In light of the unbridled, disease-promoting inflammation within the cardiovascular system, the dampened immune responses to bacterial intruders appear paradoxical at first sight. Today it is clear that innate immunity’s lowered anti-bacterial defense capabilities are significantly relayed by dysfunctional myeloid cells in CKD. The body of evidence for myeloid-mediated immunosuppression in CKD is substantial and involves polymorphonuclear leukocytes, i.e., neutrophilic granulocytes, monocytes and monocyte-derived dendritic cells.

**Fig. 4:**
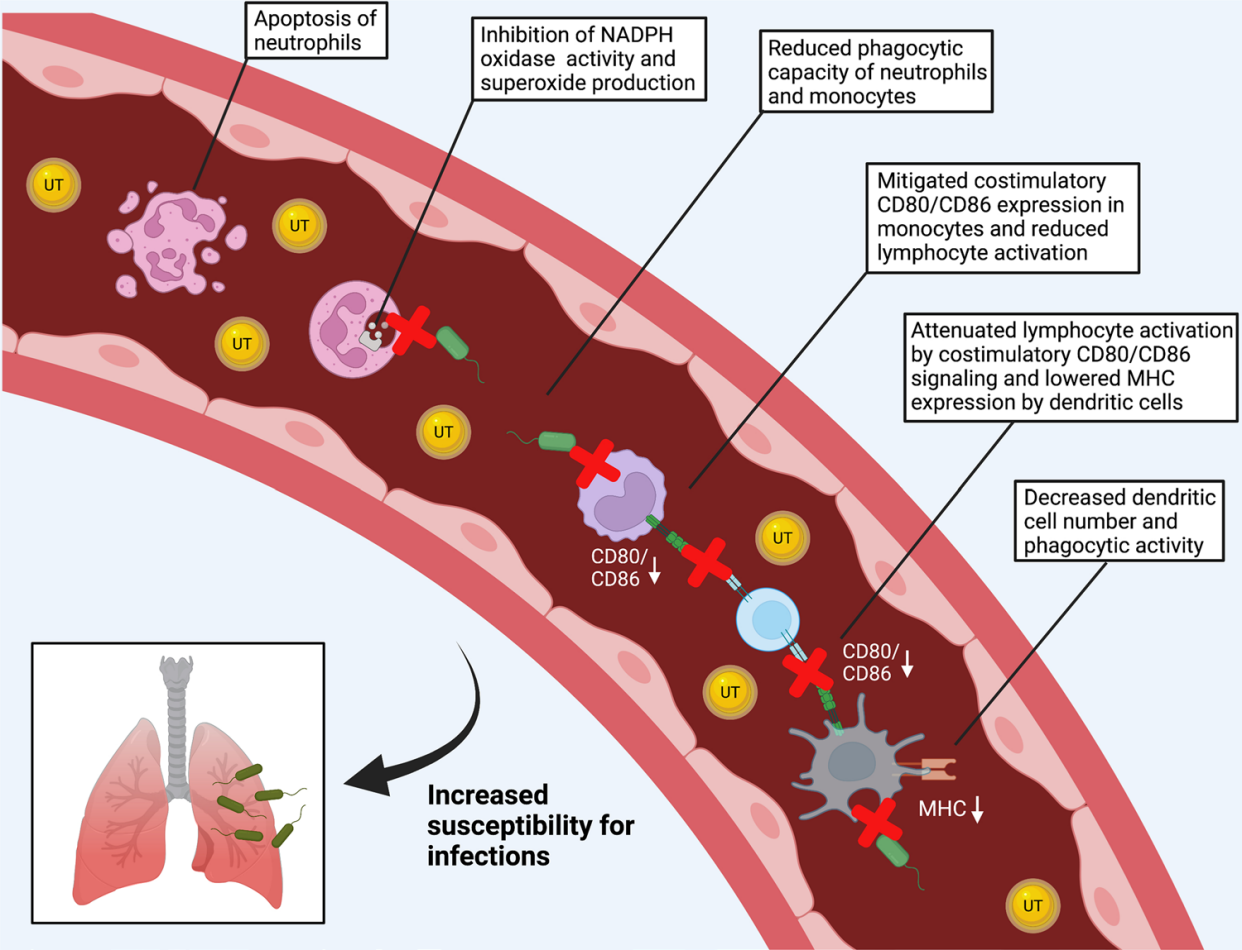
Chronic kidney disease impairs anti-bacterial host defense. Uremic toxins (UT, small yellow circles) increase the host’s susceptibility for infection via compromising proper myeloid cell function [56]. Uremic toxins promote neutrophil apoptosis and impair the cells’ phagocytic and oxidative capacity [12, 52]. Likewise, monocytes’ phagocytic activity is mitigated. Reduced co-stimulatory CD80/86 signaling hinders intact T-cell activation [76]. Under uremic conditions, dendritic cells decrease in numbers and display reduced phagocytosis and antigen presentation [136, 138]

Next to stationary or tissue-inhabiting cells, neutrophils rapidly root out and combat bacterial invaders. Equipped with various tools in their quiver, neutrophils initiate a powerful emergency response to prokaryotic infiltrators: Degranulation, extracellular traps (NETs), and phagocytosis may be employed to this end; all of which may be corrupted in CKD (Fig. 4) [52]. In-vitro, uremic plasma accelerates neutrophil apoptosis, diminishes superoxide production, and impairs phagocytosis of bacteria [12]. Impaired phagocytosis correlates inversely with the severity of uremia, and hemodialysis temporarily ameliorates phagocytic activity [79]. Following phagocytosis of bacteria, oxygen-dependent mechanisms create potent microbicidal reactive oxygen species. For instance, membrane-bound NADPH-oxidase releases superoxide anions into the phagosome, which subsequently dismutate into hydrogen peroxide aiding to neutralize engulfed microorganisms. Uremic toxins significantly interfere with the enzyme’s activity [65]. When isolated blood leukocytes were incubated with uremic retentions solutes, 39 out of the 48 examined molecules markedly diminished NADPH-oxidase activity in-vitro [121]. Likewise, uremic p-cresol suppresses NADPH-oxidase and myeloperoxidase activity at concentrations found in CKD [131]. Others showed that uremic guanidino compounds decreased superoxide formation via inhibition of glycolysis and consecutive energy depletion in neutrophils [50]. Thus, interference with reactive oxygen species generation represents a central element in CKD-mediated immunosuppression. Migration along a molecular gradient i.e. chemotaxis directs proper recruitment to the injured site. Leptin, known for its involvement in metabolism and obesity, increases in patients with CKD but significantly impairs neutrophil chemotaxis in-vitro, likely by reducing the cells’ sensitivity to chemoattractants [100]. In a small study involving 59 patients, GM-CSF treatment diminished apoptosis of neutrophils obtained from CKD patients. The authors speculate that GM-CSF in CKD may have therapeutic potential to augment immunity against infections [152].

Beside neutrophilic granulocytes, monocytes display immunosuppressive features when kidney function fails. CKD impedes monocytes’ phagocytic capacity [88]. Monocytes from patients undergoing hemodialysis exhibit reduced FITC-Dextran particle uptake compared to controls [76]. When renal replacement therapy is required, human CD14^+^ monocytes reduce surface expression of co-stimulatory CD86 (B7-2), likely impacting lymphocyte interaction [37]. The attenuated immune response may be persistent. Lipopolysaccharide (LPS)-induced expression of lymphocyte co-stimulating CD40, CD80, and CD86 remains mitigated when CD14^+^ monocytes from CKD patients are consecutively cultivated under non-uremic condition and LPS effects on co-stimulatory molecule expression remain absent in uremic media [76]. Uremia may significantly impact leukocytes’ response to viral infections with consequences for bacterial encounters. Monocytes obtained from patients undergoing hemodialysis may be more susceptible for herpes simplex virus type-2 infection. The compromised renal function goes along with a curbed cytokine production such as TNF-α, Interferon-α, and IL-12 in response to LPS in-vitro. Proper viral inhibition was only observed in monocytes obtained from healthy controls [6].

**Fig. 5:**
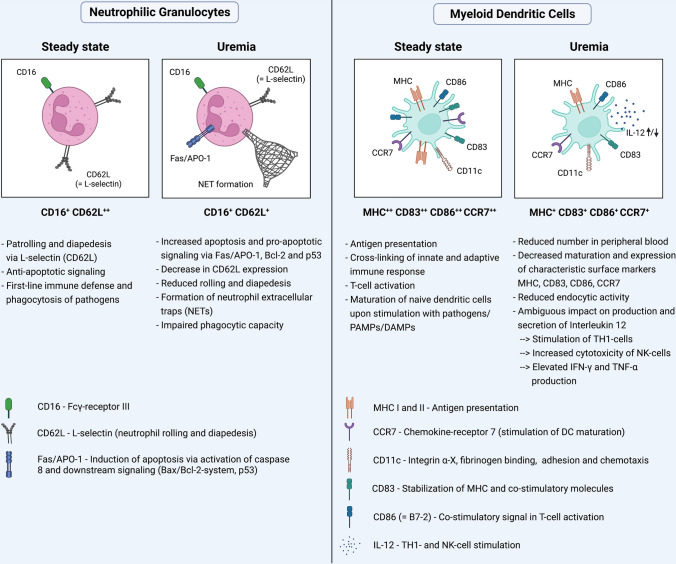
Uremia impairs neutrophil and myeloid dendritic cell function. Reduced neutrophilic CD62L expression impairs rolling and diapedesis, as assessed in hemodialysis patients [12, 64]. Activation of the Fas/APO-1 pathway induces pro-apoptotic signaling via Bax/Bcl-2 system and p53 in neutrophils isolated from uremic patients [80]. The formation of extracellular traps increases but neutrophils’ phagocytic capacity decreases in CKD [64]. Uremic toxins inhibit myeloid dendritic cell maturation, reduce expression of characteristic surface markers, and impair endocytic activity [138]. Dendritic cell numbers decrease in CKD patients’ peripheral blood [136]. CKD’ s impact on dendritic cells’ capability to secrete Interleukin-12 remains incompletely understood, as the cytokine release is increased or impaired depending on the setting [35, 76, 138]. Uremic toxin-induced IL-12 secretion, however, alters activation of natural killer (NK) cells and Th-1 lymphocytes and subsequent IFN-γ and TNF-α release [76]

Monocytes contribute to the supply of the heterogenous population of antigen-presenting dendritic cells. Dendritic cells decrease numerically in peripheral blood in patients with CKD and display reduced surface major histocompatibility complex expression [103, 137]. Both myeloid, i.e., monocyte-derived and plasmacytoid dendritic cell levels diminish, respectively, by 29 and 43%, as analyzed by flow cytometry in a study involving 245 patients with CKD stage III [103]. And when renal function is replaced by hemodialysis, dendritic cell levels are even 50% lower compared to healthy controls [136]. Ex-vivo, robust evidence suggests a prior uremic environment hampers monocyte to dendritic cell maturation and reduces characteristic dendritic cell surface markers and co-stimulatory molecules such as CD83, CD86, and CCR7 [35, 76, 138] (Fig. 5). Another small study on the contrary showed accelerated dendritic cell maturation of isolated monocytes from CKD patients [23]. Different experimental settings and varying types and amounts of uremic toxins may explain these diverging results. Likewise, CKD’s manifold effects on cytokine generation are incompletely understood. Incubation with indoxyl sulfate decreases pro-inflammatory cytokine secretion by dendritic cells [35]. Contingent on the experimental setting and uremic toxin under study, IL-12 release, for instance, may be augmented, unchanged, or even reduced [35, 76, 138]. IL-12 signaling, which promotes natural killer cell and T-cell activation, may in the aggregate be corrupted under uremic conditions in-vivo. This may conceivably relay the improper response to vaccination, which is a common phenomenon in CKD [69]. In this context, patients who did not properly responded to vaccination in a small study with 20 patients displayed a less mature dendritic cell phenotype with consequences for autologous T-cell proliferation. Interestingly, GM-CSF treatment reinstates immunity and renders primary non-responding CKD patients responsive to hepatitis B vaccination in distinct settings [59, 136].

Finally, CKD impairs myeloid dendritic cells’ endocytotic activity [76]. In light of cholesterol crystals’ inflammatory potency, it is particularly noteworthy that uremia may mitigate sterile inflammation, as uric acid crystal-induced, inflammasome-mediated gout flares attenuate when CKD progresses [3, 4, 24]. Taken together, various myeloid cells are compromised in CKD and account for the dysfunctional immune response in non-sterile but also sterile inflammation. CKD’s engendered multi-facetted immune turbulences, however, remain incompletely understood. We will contrast inflammatory and immunosuppressive mechanisms below.

## Synopsis

Whereas myeloid leukocytes' inflammatory effects promote cardiovascular disease progression, concurrent impaired innate immunity facilitates serious infections in CKD. This conundrum has not been solved, perhaps as most research focused unilaterally on one or the other entity.

### Macrophages and monocytes: engines for inflammation in CKD?

Unsurprisingly, much evidence suggests CKD-driven vascular inflammation employs monocytes and resident macrophages on a cellular level. Likewise, first data point towards a promoting role of macrophages in heart valve disease [30, 106]. An unambiguous involvement of tissue-resident macrophages in impaired immune defense on the contrary is less clear. Understanding how macrophages residing in tissues other than the vasculature or valve respond to uremia may help understand these discrepancies. Conceivably, remote tissue dysfunction in uremia involves tissue-resident macrophages in light of the profound inflammatory response in vascular tissue. Proven inter-population heterogeneity, tissue-specific transcriptional profiles, and the dictating role of macrophages’ microenvironment to injury may yet significantly determine CKD’s effect on remote tissues [31, 53, 71].

Whereas little data imply macrophages mediate the heightened susceptibility to infection, monocytes’ responses to CKD are more diverse. Studies focusing on inflammation observed heightened levels of distinct, CD14^+^ monocyte populations and augmented expression of pattern-recognition receptors [45, 66, 90, 107, 110]. CD14 binds lipopolysaccharide (LPS) complexes, TLRs sense microbial ligands and TLR-4 is the prototypical LPS responder [98, 102, 148]. While monocytes’ enhanced inflammatory status is consistent with augmented endothelial adhesiveness and generally atherogenic features, these findings seem less compatible with immunosuppression in CKD. In contrast, decreased phagocytic activity and reduced expression of surface co-stimulatory molecules may contribute to impaired immunity. Various questions arise: do monocytes exhibit both features simultaneously or do distinct monocytic subsets exist? Are inflammatory or immunosuppressive characteristics a function of CKD’s stage? Do distinct ligands i.e. uremic toxins preferentially nudge monocytes towards either response and does uremic toxin composition change over time? Because monocytes circulate, a tissue-specific effect as observed for macrophages in other settings appears unlikely. As monocytes’ disease-promoting and inflammatory role in the cardiovascular system are significant, the lowered threshold for infections may conceivably be more relevantly relayed by other immune cells.

### Neutrophils and dendritic cells: mediators of immunosuppression?

Neutrophil activation contributes to low-grade systemic inflammation in CKD and thus fosters vascular disease. Neutrophils’ impact on valvular inflammation and degeneration remains unclear. However, granulocyte subsets may accelerate cardiovascular calcification, which may also be relevant in valve disease. Studies indicate that neutrophils partake in systemic inflammation via augmented reactive oxygen production [17]. Enhanced superoxide release in uremic neutrophils can be elicited by the Protein-C kinase agonist PMA whereas use of zymosan–a molecule neutrophils avidly phagocytose– reduces superoxide release [122]. That uremic neutrophils display a reduced NADPH-oxidase activity following bacterial uptake is in line with this observation [65]. Therefore, the mechanism of activation may crucially regulate neutrophils response in the uremic microenvironment. The way neutrophils react to external stimuli may further be determined by priming. In this regard, inappropriate priming may cause an elevated baseline activity but mitigated emergency response required to repel prokaryotes [17, 122]. Further, uremic toxins may differentially influence neutrophil granulocytes, e.g., p-cresol does not affect leukocytes’ baseline oxidative burst activity as opposed to p-cresyl sulfate [119]. Overall, the current body of evidence indicates profound granulocyte dysfunction in CKD, but neutrophils’ diverse facets in this disorder remain inscrutable. Little is known about dendritic cells’ contribution to the state of immunosuppression in CKD. Uremic toxin mediated inhibition of cell maturation, decrease in numbers, reduced stimulation of the adapted immune system and presentation of antigens suggest that the aggregate response impairs immunity in CKD [35, 76, 137, 138].

### Conclusion

Macrophages, monocytes, neutrophils, and myeloid dendritic cells are decisively impacted by impaired renal function. Whereas effects on monocytes and vascular macrophages are predominantly pro-inflammatory and pro-calcific, neutrophils and myeloid dendritic cells may relevantly contribute to the state of immunosuppression. A sole dichotomous view based on cell type does not seem suitable in light of the disease’s complexity on many levels and missing knowledge such as CKD’s role in myeloid dendritic cell behavior in cardiovascular tissues. However, myeloid leukocytes’ array of different responses may in aggregate explain the concomitant existence of augmented baseline inflammation and impaired host defense. Whether the same cell type acts inflammatory while displaying reduced anti-bacterial defense capabilities is unclear. Inadequate priming of polymorphonuclear granulocytes in CKD may be one instance, in which a single cell type contributes to heightened inflammation while also displaying compromised anti-bacterial immunity. Subset heterogeneity i.e. cells of the same population exert diverging functions may be another explanation for the Janus-faced myeloid cell response in CKD. That CKD’s complications are mediated via different intracellular signaling pathways is conceivable, however, has not been systematically investigated to date.

### Future perspectives

The field of systems immunology is evolving and may help decipher CKD’s complex impact on the innate immune system. To better understand this convoluted situation, future studies should not exclusively focus on a single cell type, but instead assess differences in myeloid leukocyte responses, examine subset alterations and systemic interdependencies. Further, a more thorough and comparative analysis of the uremic microenvironment may help decipher uremic toxins’ pleiotropic effects. Profiling of uremic retention solutes have led to a comprehensive catalog of molecules, yet inter-molecule differences remain largely obscure [130]. Recently developed sequencing technologies will allow the community to obtain unprecedented insights into leukocyte subsets in CKD, and on a systems level will allow us to identify populations that either further inflammation or impair immunity. Targeting select tissues or cell types remains challenging but feasible. For instance, RNAi-mediated disruption of hepatic transthyretin production appears promising in hereditary amyloidosis [1]. Likewise, employing siRNA targeting PCSK9 proved useful against hypercholesterolemia [34]. While similar approaches to interfere with myeloid cells are technically more difficult, distinct nanoparticles are suitable vehicles to modulate gene expression in e.g. monocytes [81]. Silencing genes in myeloid cells that promote calcifying processes thus seems feasible to mitigate CKD’s systemic complications. However, a more refined understanding is a prerequisite to developing therapies that may intervene contingent on CKD’s stage and type of complication–taking leukocyte subtype and organ compartment into account.

## Supplementary Information

Below is the link to the electronic supplementary material.

Supplementary file1 (PDF 119 KB)
