# Supplementary material for: Myeloid leukocytes’ diverse effects on cardiovascular and systemic inflammation in chronic kidney disease
Source: Basic Res Cardiol. 2022 Jul 27;117(1):38. doi: 10.1007/s00395-022-00945-4 (PMC9329413; doi:10.1007/s00395-022-00945-4)
Supplement: Supplementary file 1 — Supplementary file1 (PDF 119 KB) [file 395_2022_945_MOESM1_ESM.pdf]

| | CKD | HLP | AHT | DM2 | References |
|---|---|---|---|---|---|
| **Neutrophils** | | | | | |
| Increased ROS production | ✓ | ✓ | ✓ | ✓ | PMID: 15987755, 19467225, 34148367, 33198361 |
| MPO release | ✓ | ✓ | ✓ | ✓ | PMID: 15987755, 22014237, 26223257, 23521574 |
| **Monocytes** | | | | | |
| Rise in intermediate phenotype | ✓ | ✓ | ✓ | ✗ | PMID: 21625090, 34502027, 29800237 |
| Augmented adhesion and migration | ✓ | ✓ | ✓ | ✓ | PMID: 12697729, 25830914 18788855, 12117736, 17395876 |
| Elevated TLR2/4 expression | ✓ | ✓ | ✓ | ✓ | PMID: 20729266, 29481790 20959532, 29622775, 22533660 |
| Activation of Wnt/β-catenin-pathway | ✓ | ✗ | ✗ | ✗ | PMID: 23935909 |
| Reduced expression of calcium-sensing receptor | ✓ | ✗ | ✗ | ✗ | PMID: 33647324 |
| **Macrophages** | | | | | |
| FGF23- induced proliferation and TNF-α production | ✓ | ✗ | ✗ | ✗ | PMID: 25739891, 22714041 |
| Proinflammatory cytokine release | ✓ | ✓ | ✓ | ✓ | PMID: 30586693, 30940651 20959532, 29622775 |

**Suppl. Table 1: Shared effects of CKD and other cardiovascular risk factors on myeloid cell function and characteristics.** Whereas some of the myeloid cell alterations outlined in figure 2 have been only described in CKD, others effects overlap with established cardiovascular risk factors e.g. hyperlipidemia (HLP), arterial hypertension (AHT) and diabetes mellitus type 2 (DM2). Checkmarks indicate overlapping features. References [24] [25] [9] [2] [18] [5] [21] [19] [26] [12] [3] [23] [6] [22] [20] [10] [7] [4] [8] [13] [1] [14] [15] [11] [16] [17] are provided in the right column.


1. Al-Chaqmaqchi, H. A., Moshfegh, A., Dadfar, E., Paulsson, J., Hassan, M., Jacobson, S. H., & Lundahl, J. (2013). Activation of Wnt/β-catenin pathway in monocytes derived from chronic kidney disease patients. *PLoS One*, *8*(7), e68937. doi:10.1371/journal.pone.0068937
2. Araos, P., Figueroa, S., & Amador, C. A. (2020). The Role of Neutrophils in Hypertension. *Int J Mol Sci*, *21*(22), E8536. doi:10.3390/ijms21228536
3. Cybulsky, M. I., & Hegele, R. A. (2003). The fractalkine receptor CX3CR1 is a key mediator of atherogenesis. *J Clin Invest*, *111*(8), 1118-1120. doi:10.1172/JCI18237
4. Dasu, M. R., & Jialal, I. (2011). Free fatty acids in the presence of high glucose amplify monocyte inflammation via Toll-like receptors. *Am J Physiol Endocrinol Metab*, *300*(1), E145-54. doi:10.1152/ajpendo.00490.2010
5. Derosa, G., Mugellini, A., Pesce, R. M., D'Angelo, A., & Maffioli, P. (2015). A study about the relevance of adding acetylsalicylic acid in primary prevention in subjects with type 2 diabetes mellitus: effects on some new emerging biomarkers of cardiovascular risk. *Cardiovasc Diabetol*, *14*, 95. doi:10.1186/s12933-015-0254-8
6. Devaraj, S., & Jialal, I. (2008). Validation of the circulating monocyte being representative of the cholesterol-loaded macrophage: biomediator activity. *Arch Pathol Lab Med*, *132*(9), 1432-1435. doi:10.5858/2008-132-1432-VOTCMB

7. Grabulosa, C. C., Manfredi, S. R., Canziani, M. E., Quinto, B. M. R., Barbosa, R. B., Rebello, J. F., Batista, M. C., Cendoroglo, M., & Dalboni, M. A. (2018). Chronic kidney disease induces inflammation by increasing Toll-like receptor-4, cytokine and cathelicidin expression in neutrophils and monocytes. *Exp Cell Res*, *365*(2), 157-162. doi:10.1016/j.yexcr.2018.02.022
8. Gupta, S., Maratha, A., Siednienko, J., Natarajan, A., Gajanayake, T., Hoashi, S., & Miggin, S. (2017). Analysis of inflammatory cytokine and TLR expression levels in Type 2 Diabetes with complications. *Sci Rep*, *7*(1), 7633. doi:10.1038/s41598-017-07230-8
9. Johnson, J., Jaggers, R. M., Gopalkrishna, S., Dahdah, A., Murphy, A. J., Hanssen, N. M. J., & Nagareddy, P. R. (2022). Oxidative Stress in Neutrophils: Implications for Diabetic Cardiovascular Complications. *Antioxid Redox Signal*, *36*(10-12), 652-666. doi:10.1089/ars.2021.0116
10. Koc, M., Toprak, A., Arikan, H., Odabasi, Z., Elbir, Y., Tulunay, A., Asicioglu, E., Eksioglu-Demiralp, E., Glorieux, G., Vanholder, R., & Akoglu, E. (2011). Toll-like receptor expression in monocytes in patients with chronic kidney disease and haemodialysis: relation with inflammation. *Nephrol Dial Transplant*, *26*(3), 955-963. doi:10.1093/ndt/gfq500
11. Komaba, H., & Fukagawa, M. (2012). The role of FGF23 in CKD--with or without Klotho. *Nat Rev Nephrol*, *8*, 484-490. doi:10.1038/nrneph.2012.116
12. Loperena, R., Van Beusecum, J. P., Itani, H. A., Engel, N., Laroumanie, F., Xiao, L., Elijovich, F., Laffer, C. L., Gnecco, J. S., Noonan, J., Maffia, P., Jasiewicz-Honkisz, B., Czesnikiewicz-Guzik, M., Mikolajczyk, T., Sliwa, T., Dikalov, S., Weyand, C. M., Guzik, T. J., & Harrison, D. G. (2018). Hypertension and increased endothelial mechanical stretch promote monocyte differentiation and activation: roles of STAT3, interleukin 6 and hydrogen peroxide. *Cardiovasc Res*, *114*(11), 1547-1563. doi:10.1093/cvr/cvy112
13. Marketou, M. E., Kontaraki, J. E., Zacharis, E. A., Kochiadakis, G. E., Giaouzaki, A., Chlouverakis, G., & Vardas, P. E. (2012). TLR2 and TLR4 gene expression in peripheral monocytes in nondiabetic hypertensive patients: the effect of intensive blood pressure-lowering. *J Clin Hypertens (Greenwich)*, *14*(5), 330-335. doi:10.1111/j.1751-7176.2012.00620.x
14. Mary, A., Objois, T., Brazier, M., Bennis, Y., Boudot, C., Lenglet, G., Paccou, J., Bugnicourt, J. M., Choukroun, G., Drueke, T. B., Massy, Z. A., Kamel, S., Six, I., & Mentaverri, R. (2021). Decreased monocyte calcium sensing receptor expression in patients with chronic kidney disease is associated with impaired monocyte ability to reduce vascular calcification. *Kidney Int*, *99*(6), 1382-1391. doi:10.1016/j.kint.2021.01.026
15. Masuda, Y., Ohta, H., Morita, Y., Nakayama, Y., Miyake, A., Itoh, N., & Konishi, M. (2015). Expression of Fgf23 in activated dendritic cells and macrophages in response to immunological stimuli in mice. *Biol Pharm Bull*, *38*(5), 687-693. doi:10.1248/bpb.b14-00276
16. Nakano, T., Katsuki, S., Chen, M., Decano, J. L., Halu, A., Lee, L. H., Pestana, D. V. S., Kum, A. S. T., Kuromoto, R. K., Golden, W. S., Boff, M. S., Guimaraes, G. C., Higashi, H., Kauffman, K. J., Maejima, T., Suzuki, T., Iwata, H., Barabási, A. L., Aster, J. C., Anderson, D. G., Sharma, A., Singh, S. A., Aikawa, E., & Aikawa, M. (2019). Uremic Toxin Indoxyl Sulfate Promotes Proinflammatory Macrophage Activation Via the Interplay of OATP2B1 and Dll4-Notch Signaling. *Circulation*, *139*(1), 78-96. doi:10.1161/CIRCULATIONAHA.118.034588
17. Opdebeeck, B., Maudsley, S., Azmi, A., De Maré, A., De Leger, W., Meijers, B., Verhulst, A., Evenepoel, P., D'Haese, P. C., & Neven, E. (2019). Indoxyl Sulfate and p-

Cresyl Sulfate Promote Vascular Calcification and Associate with Glucose Intolerance. *J Am Soc Nephrol*, *30*(5), 751-766. doi:10.1681/ASN.2018060609
18. Puntoni, M., Sbrana, F., Bigazzi, F., Minichilli, F., Ferdeghini, E., & Sampietro, T. (2011). Myeloperoxidase modulation by LDL apheresis in familial hypercholesterolemia. *Lipids Health Dis*, *10*, 185. doi:10.1186/1476-511X-10-185
19. Ramírez, R., Carracedo, J., Merino, A., Soriano, S., Ojeda, R., Alvarez-Lara, M. A., Martín-Malo, A., & Aljama, P. (2011). CD14+CD16+ monocytes from chronic kidney disease patients exhibit increased adhesion ability to endothelial cells. *Contrib Nephrol*, *171*, 57-61. doi:10.1159/000327134
20. Riou, S., Mees, B., Esposito, B., Merval, R., Vilar, J., Stengel, D., Ninio, E., van Haperen, R., de Crom, R., Tedgui, A., & Lehoux, S. (2007). High pressure promotes monocyte adhesion to the vascular wall. *Circ Res*, *100*(8), 1226-1233. doi:10.1161/01.RES.0000265231.59354.2c
21. Rovira-Llopis, S., Rocha, M., Falcon, R., de Pablo, C., Alvarez, A., Jover, A., Hernandez-Mijares, A., & Victor, V. M. (2013). Is myeloperoxidase a key component in the ROS-induced vascular damage related to nephropathy in type 2 diabetes. *Antioxid Redox Signal*, *19*(13), 1452-1458. doi:10.1089/ars.2013.5307
22. Sampson, M. J., Davies, I. R., Brown, J. C., Ivory, K., & Hughes, D. A. (2002). Monocyte and neutrophil adhesion molecule expression during acute hyperglycemia and after antioxidant treatment in type 2 diabetes and control patients. *Arterioscler Thromb Vasc Biol*, *22*(7), 1187-1193. doi:10.1161/01.atv.0000021759.08060.63
23. Schepers, E., Houthuys, E., Dhondt, A., De Meyer, G., Neirynck, N., Bernaert, P., Van den Bergh, R., Brouckaert, P., Vanholder, R., & Glorieux, G. (2015). Transcriptome analysis in patients with chronic kidney disease on hemodialysis disclosing a key role for CD16+CX3CR1+ monocytes. *PLoS One*, *10*(4), e0121750. doi:10.1371/journal.pone.0121750
24. Sela, S., Shurtz-Swirski, R., Cohen-Mazor, M., Mazor, R., Chezar, J., Shapiro, G., Hassan, K., Shkolnik, G., Geron, R., & Kristal, B. (2005). Primed peripheral polymorphonuclear leukocyte: a culprit underlying chronic low-grade inflammation and systemic oxidative stress in chronic kidney disease. *J Am Soc Nephrol*, *16*(8), 2431-2438. doi:10.1681/ASN.2004110929
25. Vasconcelos, E. M., Degasperi, G. R., de Oliveira, H. C., Vercesi, A. E., de Faria, E. C., & Castilho, L. N. (2009). Reactive oxygen species generation in peripheral blood monocytes and oxidized LDL are increased in hyperlipidemic patients. *Clin Biochem*, *42*(12), 1222-1227. doi:10.1016/j.clinbiochem.2009.05.010
26. Williams, H., Mack, C. D., Li, S. C. H., Fletcher, J. P., & Medbury, H. J. (2021). Nature versus Number: Monocytes in Cardiovascular Disease. *Int J Mol Sci*, *22*(17), 9119. doi:10.3390/ijms22179119
